# Job Satisfaction Among Healthcare Providers in a Tertiary Care Government Medical College and Hospital in Chhattisgarh

**DOI:** 10.7759/cureus.41111

**Published:** 2023-06-28

**Authors:** Namita Deshmukh, Purnima Raj, Pratik Chide, Avinash Borkar, Gajanan Velhal, Rahul Chopade

**Affiliations:** 1 Department of Community Medicine, Bhaktshreshtha Kamalakarpant Laxman (BKL) Walawalkar Rural Medical College, Ratnagiri, IND; 2 Department of Pharmacology, Late Shri Lakhiram Agrawal Memorial Medical College, Raigarh, IND; 3 Department of Pathology, Government Medical College, Miraj, IND

**Keywords:** job satisfaction, global healthcare systems, healthcare provider, job security, job satisfaction scale

## Abstract

Background

The job satisfaction of healthcare providers is particularly relevant in service management because employees’ level of job satisfaction impacts healthcare service users. A positive association is observed between the job satisfaction of healthcare providers and patient satisfaction. This study was conducted to determine the job satisfaction levels and their determinants among healthcare providers (doctors, nurses, and support staff) in a tertiary care hospital.

Methodology

A cross-sectional study was conducted among 400 healthcare providers of a tertiary care medical college and hospital. The questionnaire method was employed to collect demographic data. Job satisfaction level was assessed using the Job Satisfaction Survey Scale (JSS Scale).

Results

The mean overall satisfaction score among doctors was 123.05 ± 17.06, for nurses 127.4 ± 14.58, and for the support staff 138.46 ± 22.09. Among all three groups, employees’ support staff were found to be more satisfied (40%), followed by doctors (15%) and nurses (6.67%), while the proportion of dissatisfied employees was highest among doctors (20%) than nurses (6.67%) and the support staff (6.67%). Satisfaction was significantly higher among the support staff compared to doctors and nurses. The level of satisfaction was found to be more in the younger staff (38.14%), regular job holders (33.33%), and fresh recruits (37.5%). Overall, satisfaction levels were found to be positively associated with factors such as the type and nature of work (64%) and relationships with co-workers (40%) and supervisors (36%) while more dissatisfied with factors such as interpersonal communication (70%), salary (54%), and promotional opportunities (42%).

Conclusion

The overall satisfaction among employees was only 20%. Factors such as job security, young age, and work experience were strongly associated with job satisfaction. Employees were more satisfied with the type and nature of work and relationships with co-workers while more dissatisfied with salary, promotional opportunities, and interpersonal communication.

## Introduction

The concept of job satisfaction is relevant even today; it means an individual’s positive emotional reaction and attitude toward his job. Many theories explain the term “job satisfaction” and describe the factors and determinants responsible for generating this positive emotion. A recent study demonstrates that the important determinants leading to job satisfaction are interesting job/work, good relationships with seniors and colleagues, high income, being allowed to work independently, and having clearly defined career advancement opportunities [1-3].

It is widely accepted that social and environmental factors contribute to the occurrence of many diseases in humans. As the job/profession is the single most important activity of any individual, pressure and stress at the workplace directly impact the physical and mental health of an employee and determine the quality of his or her work life. A happy worker tends to be more efficient and productive and may apply innovative ways of doing his or her job better. In contrast, a dissatisfied worker tends to be bored and unhappy and may procrastinate, which can negatively impact the structure and workflow of the organization for which he or she is working [1,4,5]. As a service industry, healthcare primarily focuses on human interactions and infrastructure. At any point in healthcare delivery, a team is involved. Building motivation in the team members to perform and work for the betterment of patients is an important task performed by every doctor, apart from using his or her knowledge and skills in treating patients and day-to-day management. As a team leader, a healthcare worker’s job satisfaction is vital to overall team performance, patient outcome, and patient satisfaction [6]. If the leader feels dissatisfied, he or she will generate sub-optimal healthcare providers and poor clinical outcomes. For the healthcare worker, sub-optimal healthcare and poor clinical outcomes may add to his or her stress and burnout, resulting in more dissatisfaction [7,8].

In a teaching hospital, where doctors and nurses play the twin role of teaching and patient care, this might have wider ramifications, as they shoulder the responsibility for public health and shape the attitudes and aptitudes of budding doctors and nurses [9,10].

The job satisfaction of medical experts and health workers is a significant phenomenon of research to strengthen the healthcare system that will benefit the mass population. Hence, the current study aims at studying the job satisfaction levels of healthcare providers at a tertiary medical college and hospital.

## Materials and methods

Study setting and study design

A cross-sectional study for a period of one year was conducted to describe the opinion, attitudes, and feelings toward job satisfaction among healthcare providers working at Government Medical College in Chhattisgarh, India (doctors/physicians, nurses, and the support staff, including pharmacists, laboratory assistants, laboratory technicians, social workers, stenographers, and record clerks). The employees who gave their consent were included in the study. The ethical approval for this research was obtained from the institutional ethics committee (IEC/88/GMCCH/21).

Sample size calculation and sampling technique

The sample size was determined using the following formula: n=z2p(1-p)/e2, where “z” is the standard normal deviate at a 95% confidence interval (CI), “p” is the prevalence of satisfaction among healthcare providers, and “e” is the allowable error. The value for “p” was taken from previously conducted studies as 41% [5,6]. The allowable error “e” was taken as 5%. The required sample size calculated using the above parameters was 387.

Data collection

The data was collected from 400 healthcare providers, including 160 doctors, 120 nurses, and 120 supporting employees, using a pretested and structured questionnaire. Purposive sampling was used to incorporate all cadres of healthcare workers. The first part of the questionnaire includes sociodemographic characteristics and occupation details, whereas the second part includes the Job Satisfaction Survey Scale (JSS Scale). The participants were well informed regarding the purpose of the study, and their consent was taken before administering the questionnaire. Approval was sought from the institutional ethics committee before starting the study.

The Job Satisfaction Survey Scale (JSS Scale) was used to measure job satisfaction among healthcare workers (Cronbach’s alpha: 0.91) [11]. The scale is in English and Hindi languages. We used the Hindi scale for those who did not understand English. It is a 36-item scale classified into nine facets (subscale) to assess employee attitudes and aspects of the job. The nine facets are pay, promotion, supervision, fringe benefits, contingent rewards (performance-based rewards), operating procedures (required rules and procedures), co-workers, nature of work, and communication. Each facet is assessed with four items, and a total score is computed from all items. A summated rating scale format is used, with six choices per item ranging from “strongly disagree” to “strongly agree” and given a score of 1 to 6. Scores on each of the nine facet subscales, based on four items each, can range from 4 to 24, while scores for total job satisfaction, based on the sum of all 36 items, can range from 36 to 216.

Analysis and Interpretation of Scale

The JSS Scale determines job satisfaction as dissatisfied and satisfied. For the four-item subscale, for total scores from 4 to 24, scores of 4-12 indicate dissatisfaction, 13-16 indicate ambivalent, and 17-24 indicate satisfaction. For the 36-item subscale, for total scores from 36 to 21, scores of 36-108 indicate dissatisfaction, 109-144 indicate ambivalent, and 145-216 indicate satisfaction.

Data analysis and statistical techniques

Data were analyzed using the Statistical Package for the Social Sciences (SPSS-16) (IBM SPSS Statistics, Armonk, NY, USA) trial version. Continuous variables were summarized in terms of means and standard deviations, while categorical variables were in frequencies and percentages. Statistical analysis was done by percentages and using the chi-square test. The statistical significance of differences between groups was tested, and a p-value of <0.05 was taken as statistically significant.

## Results

Out of 400 respondents, 160 (40%) were doctors, 120 (30%) were nurses, and 120 (30%) were support staff, including laboratory technicians, pharmacists, record clerks, and medical social workers. The mean age of the respondents was 33.1 ± 5.57 years (range: 25-50 years). About 136 (34%) were male employees, and 264 (66%) were female employees. The mean age for males was 34.35 ± 7.01 years, and the mean age for females was 32.45 ± 6.76 years. Regarding experience, 200 (50%) had less than two years of experience, 56 (14%) were with 2-5 years of experience, and the remaining 144 (36%) were with more than five years of experience in their job. A total of 264 (66%) employees were on regular posts, while 136 (34%) were on contractual (tenure of one year) (Table 1).

**Table 1:** Occupation-wise distribution of respondents

Demographic characteristics	Doctors (160)	Nurses (120)	Supportive staff (120)
Age in years			
21-30	24	56	56
31-40	96	56	64
41-50	40	8	0
Gender			
Males	56	-	80
Females	104	120	40
Experience			
Below 2 years	56	28	48
2-5 years	32	48	44
Above 5 years	72	44	28
Post			
Regular	24	120	120
Contractual	136	0	0

Out of all participants in the study, 80 (20%) were satisfied, and 48 (12%) were dissatisfied. The maximum number of employees, 272 (68%), were in the ambivalent group, which is in between satisfied and dissatisfied. Among all three groups, the support staff was more satisfied (40%), followed by doctors (15%) and nurses (6.67%), while the proportion of dissatisfied employees was highest among doctors (20%) than nurses (6.67%) and the support staff (6.67%). Satisfaction was significantly higher among the support staff as compared to doctors and nurses (χ2 = 19.02, p= 0.0001, df 1). The mean overall satisfaction score for all 36 items among doctors was 123.05 ± 17.06, among nurses 127.4 ± 14.58, and among support staff 138.46 ± 22.09. It was highest for the support staff, followed by nurses, and lowest for doctors. Thus, the proportion of satisfaction and mean overall satisfaction score were highest among the support staff (Table 2).

**Table 2:** Satisfaction among healthcare providers

Healthcare providers	Satisfied (score: 145-216)		Dissatisfied (score: 36-108)		Ambivalent (score: 109-144)	
	Number	%	Number	%	Number	%
Doctors (160)	24	15	32	20	104	65
Nurses (120)	8	6.67	8	6.67	104	86.66
Support staff (120)	48	40	8	6.67	64	53.33
Total (400)	80	20	48	12	272	68

Younger employees (≤30 years) were more satisfied (38.24%) as compared to those who are >30 years. Satisfaction was more (33.33%) among those on regular posts, and dissatisfaction was highest (23.53%) among contractual ones, which was significant. Newly recruited employees (experience: <5 years) were more satisfied (37.5%) than those with experience of >5 years (13.89%), which was highly significant. Regular employees were satisfied significantly more (33.33%) than those on contract jobs (20.59%) (Table 3).

**Table 3:** Distribution of job satisfaction according to different demographic characteristics

Variable		Total respondents	Satisfied (%)	Dissatisfied (%)	Ambivalent (%)	Df 1	X^2^	p-value
Age	≤30 years	136	52 (38.24)	20 (14.70)	64 (47.06)	6.22	0.0126	Significant
	>30 years	264	32 (12.12)	64 (24.24)	168 (63.64)			
Gender	Males	136	40 (29.41)	16 (11.76)	80 (58.82)	0.42	0.5153	Non-significant
	Females	264	40 (15.15)	32 (12.12)	192 (72.72)			
Experience	Below 5 years	256	96 (37.5)	40 (15.62)	120 (46.87)	7.83	0.0051	Highly significant
	Above 5 years	144	20 (13.89)	48 (33.33)	76 (52.78)			
Post	Regular	264	88 (33.33)	20 (7.57)	156 (59.09)	5.47	0.0194	Significant
	Contractual	136	28 (20.59)	32 (23.53)	76 (55.88)			

Out of all participants, only 56 (14%) healthcare providers were satisfied with their salaries, while more than half (216, 54%) of the employees were dissatisfied with respect to their salaries. Around 168 (42%) employees were unaware of the promotional opportunities they would get in this institution. Only 112 (28%) employees were satisfied with whatever benefits they received here. Only 96 (24%) employees thought they got any appraisal for good work. Nearly 42% of employees were not satisfied with the working environment of the hospital. A total of 160 (40%) employees were happy with their relationship with co-workers, but 280 (70%) found it difficult to communicate either with patients or others. About 36% of employees were satisfied with their relationship with supervisors, and 64% were satisfied with the nature and type of work they do. The mean satisfaction score for the four-item subscale was maximum (18.44 ± 3.80) for the nature of work and was the only score that fell under the satisfaction category (17-24). The mean score was minimum (11.88 ± 3.89) for the pay subscale and was the only score that fell under the dissatisfaction category. The mean satisfaction score for all other subscales (promotion, supervision, fringe benefits, contingent rewards, operating procedures, co-workers, and communication) falls under the ambivalent category (Table 4).

**Table 4:** Job satisfaction for the four-item subscales for all participants (N=400)

Serial number	Four-item subscales	Satisfaction		Dissatisfaction		Ambivalent	
		Number	%	Number	%	Number	%
1	Pay	56	14	216	54	128	32
2	Promotion	56	14	168	42	176	44
3	Supervision	144	36	56	14	200	50
4	Fringe benefits	112	28	176	44	112	28
5	Contingent rewards	96	24	152	38	152	38
6	Operating procedures	56	14	168	42	176	44
7	Co-workers	160	40	40	10	200	50
8	Nature of work	256	64	16	4	128	32
9	Communication	64	16	280	70	56	14

More doctors were dissatisfied with communication with people (66.67%), followed by salary (53.33%) and benefits received (53.33%). More nurses were dissatisfied with their salary (73.34%), followed by promotion opportunities (46.67%), while more support staff were dissatisfied with communication with people (66.67%), followed by salary (53.33%). Overall, salary was the common factor for dissatisfaction among all three groups. Less than 7% of employees in all three groups quoted the nature of work, and only nurses (33,33 %) quoted relationships with other workers and supervision as factors of dissatisfaction (Table 5).

**Table 5:** Comparison between the three groups of healthcare providers’ attitudes toward job satisfaction

Serial number	Subscales	Dissatisfaction					
		Doctors (160)		Nurses (120)		Support staff (120)	
		Number	%	Number	%	Number	%
1	Pay	64	53.33	88	73.34	64	53.33
2	Promotion	56	46.67	56	46.67	56	46.67
3	Supervision	8	6.67	40	33.33	8	6.67
4	Fringe benefits	64	53.33	48	40	64	53.33
5	Contingent rewards	56	46.67	40	33.33	56	46.67
6	Operating procedures	56	46.67	56	46.67	56	46.67
7	Co-workers	0	0	40	33.33	0	0
8	Nature of work	8	6.67	0	0	8	6.67
9	Communication	80	66.67	40	33.33	80	66.67

On comparing mean satisfaction scores among all three groups for the four-item subscales, the mean score was the maximum for the nature of work in all three groups (16.65, 18.66, and 20 for doctors, nurses, and support staff, respectively). This depicts that all employees were satisfied with the type of work they do at the hospital. It was followed by co-workers. This indicates that the relationship between co-workers and colleagues was a healthy one (Table 6).

**Table 6:** Comparison of mean satisfaction score among all three groups (for the four-item subscales)

Serial number	Subscales	Mean score		
		Doctors	Nurses	Support staff
1	Pay	13.2	10.2	12.26
2	Promotion	11.75	13.53	12.93
3	Supervision	12.75	13.66	19
4	Fringe benefits	14.45	14.06	12.4
5	Contingent rewards	13.6	13.86	14.26
6	Operating procedures	12.6	13.86	12.26
7	Co-workers	14.4	15.26	19
8	Nature of work	16.65	18.66	20
9	Communication	13.65	13.6	12.06

## Discussion

This study found that 20% of employees were satisfied, while 12% were dissatisfied. The majority of respondents (68%) answered the questions that fall in the ambivalent category, i.e., they were not satisfied or dissatisfied. Overall satisfaction was highest among the support staff (40%), followed by doctors (20%) and nurses (6.67%). Abate and Mekonnen found 41% satisfaction in their systematic review [12]. However, Jaiswal et al. found more job satisfaction among nurses (0.68), followed by doctors and support staff [13]. Dissatisfaction was highest among doctors (20%), consistent with the result of Kumar et al. (21%) [14], while Bhattacherjee et al. [15] found 40% dissatisfaction.

The mean satisfaction score for all 36 items was also highest for the support staff (138.46), followed by the nurses (127.4) and the doctors (123.05). The higher satisfaction among the support staff may be due to permanent jobs. According to Herzberg’s motivator-hygiene theory, job security is an important determinant of job satisfaction [8]. Employees who feel their future is secure in an organization work better than those who feel insecure about it [10]. In our study, we found that permanent employees and fresh recruits were more satisfied. Similar results were found by Bhattacherjee et al. [15]. However, Gedam et al. [16] found more satisfaction among senior health professionals.

In this study, it was found that more than 50% of healthcare professionals were dissatisfied with salary and interpersonal communication, which is similar to the study done by Senbounsou et al. [6], Bajpai [10], Jaiswal et al. [13], Bhattacherjee et al. [15], Tasneem et al. [17], and Kim et al. [18]. The salary of healthcare professionals was not according to their expectations, which can be a constraint for their productivity and hard work in the long run. Healthy communication and a positive environment are also essential among peers at different hierarchical levels [10]. More than 40% of healthcare professionals were dissatisfied with promotion (42%), recognition (44%), and working environment (42%). These results are comparable with the results of the studies done by Bajpai [10], Bhattacherjee et al. [15], and Tasneem et al. [17]. Incentives, rewards, and recognitions are the primary factors that influence job satisfaction [10]. The study by Dieleman et al. [19] suggested a positive correlation between incentives, rewards, recognition, and job satisfaction. Satisfaction was more related to the nature of work, i.e., the work assigned to them (64%), followed by a relationship with co-workers (40%). This is good for the institution because employees were happy with the opportunity to utilize their skills and talents. According to Pestonjee and Mishra [20], a working environment where people lack trust in their co-workers may result in worse organizational performance.

Recommendations

Job satisfaction of employees is one of the major factors determining the achievement of the objectives of the institution. Studies related to job satisfaction of healthcare providers in a government hospital were not conducted previously in this region. Therefore, the results of the present study will help in formulating good practices and policies for creating a better environment. The governing authority of the hospital should try to satisfy the employees by providing adequate facilities. Monetary benefits such as financial grants should be increased, and a greater number of rewards and incentives should be given to employees for higher job satisfaction levels.

Limitations of the study

We obtained more results in the ambivalent category; the participants avoided giving extreme responses, which is human behavior. We also studied only a few parameters (experience, gender, and post (regular and contractual)) related to job satisfaction.

## Conclusions

Overall, job satisfaction among healthcare providers in the current study was 20%. The support staff was comparatively more satisfied than doctors and nurses. Job security and work experience were found to be the factors that influence job satisfaction. Overall, the medical college and hospital employees were more satisfied with the type and nature of the work, their relationship with co-workers, and interpersonal communication, while more dissatisfied with salary, promotional opportunities, and the working environment.

## Appendices

Questionnaire

Proforma for the Project: Job Satisfaction Among Healthcare Providers in a Tertiary Care Government Medical College and Hospital

Age:                             Gender: Male/Female                   Occupation/designation:

Department:                                                                        Experience in this occupation:

Contractual/regular:

Job Satisfaction Survey

Please circle one number for each question that comes closest to reflecting your opinion about it: Disagree very much = 1, Disagree moderately = 2, Disagree slightly = 3, Agree slightly = 4, Agree moderately = 5, Agree very much = 6.

1. I feel I am being paid a fair amount for the work I do.

         1     2     3     4    5     6

2. There is really too little chance for promotion in my job.

         1     2     3    4    5     6

3. My supervisor is quite competent in doing his/her job.

         1     2     3    4    5     6

4. I am not satisfied with the benefits I receive.

         1     2     3    4    5     6

5. When I do a good job, I receive the recognition for it that I should receive.

         1     2     3    4    5     6

6. Many of our rules and procedures make doing a good job difficult.

         1     2     3    4    5     6

7. I like the people I work with.

         1     2     3    4    5     6

8. I sometimes feel my job is meaningless.

         1     2     3    4    5     6

9. Communications seem good within this organization.

         1     2     3    4    5     6

10. Raises are too few and far between.

         1     2     3    4    5     6

11. Those who do well on the job stand a fair chance of being promoted.

         1     2     3    4    5     6

12. My supervisor is unfair to me.

         1     2     3    4    5     6

13. The benefits we receive are as good as most other organizations offer.

         1     2     3    4    5     6

14. I do not feel that the work I do is appreciated.

         1     2     3    4    5     6

Job satisfaction survey proforma 1 and 2 are presented in Table 7 and Table 8, respectively.

**Table 7:** Proforma 1

Job Satisfaction Survey		
	Please circle one number for each question that comes closest to reflecting your opinion about it.	Disagree very much = 1, Disagree moderately = 2, Disagree slightly = 3, Agree slightly = 4, Agree moderately = 5, Agree very much = 6
1	I feel I am being paid a fair amount for the work I do.	1 2 3 4 5 6
2	There is really too little chance for promotion in my job.	1 2 3 4 5 6
3	My supervisor is quite competent in doing his/her job.	1 2 3 4 5 6
4	I am not satisfied with the benefits I receive.	1 2 3 4 5 6
5	When I do a good job, I receive the recognition for it that I should receive.	1 2 3 4 5 6
6	Many of our rules and procedures make doing a good job difficult.	1 2 3 4 5 6
7	I like the people I work with.	1 2 3 4 5 6
8	I sometimes feel my job is meaningless.	1 2 3 4 5 6
9	Communications seem good within this organization.	1 2 3 4 5 6
10	Raises are too few and far between.	1 2 3 4 5 6
11	Those who do well on the job stand a fair chance of being promoted.	1 2 3 4 5 6
12	My supervisor is unfair to me.	1 2 3 4 5 6
13	The benefits we receive are as good as most other organizations offer.	1 2 3 4 5 6
14	I do not feel that the work I do is appreciated.	1 2 3 4 5 6

**Table 8:** Proforma 2

Job Satisfaction Survey (Continuation)		
	Please circle one number for each question that comes closest to reflecting your opinion about it.	Disagree very much = 1, Disagree moderately = 2, Disagree slightly = 3, Agree slightly = 4, Agree moderately = 5, Agree very much = 6
15	My efforts to do a good job are seldom blocked by red tape.	1 2 3 4 5 6
16	I find that I have to work harder at my job because of the incompetence of the people I work with.	1 2 3 4 5 6
17	I like doing the things I do at work.	1 2 3 4 5 6
18	The goals of this organization are not clear to me.	1 2 3 4 5 6
19	I feel unappreciated by the organization when I think about what they pay me.	1 2 3 4 5 6
20	People get ahead as fast here as they do in other places.	1 2 3 4 5 6
21	My supervisor shows too little interest in the feelings of subordinates.	1 2 3 4 5 6
22	The benefit package we have is equitable.	1 2 3 4 5 6
23	There are few rewards for those who work here.	1 2 3 4 5 6
24	I have too much to do at work.	1 2 3 4 5 6
25	I enjoy my co-workers.	1 2 3 4 5 6
26	I often feel that I do not know what is going on with the organization.	1 2 3 4 5 6
27	I feel a sense of pride in doing my job.	1 2 3 4 5 6
28	I feel satisfied with my chances for salary increases.	1 2 3 4 5 6
29	There are benefits we do not have that we should have.	1 2 3 4 5 6
30	I like my supervisor.	1 2 3 4 5 6
31	I have too much paperwork.	1 2 3 4 5 6
32	I don't feel my efforts are rewarded the way they should be.	1 2 3 4 5 6
33	I am satisfied with my chances for promotion.	1 2 3 4 5 6
34	There is too much bickering and fighting at work.	1 2 3 4 5 6
35	My job is enjoyable.	1 2 3 4 5 6
36	Work assignments are not fully explained.	1 2 3 4 5 6
